# Clinicopathological predictive factors in long‐term survivors who underwent surgery for pancreatic ductal adenocarcinoma: A single‐center propensity score matched analysis

**DOI:** 10.1002/wjs.12397

**Published:** 2024-11-14

**Authors:** Carlo Ingaldi, Vincenzo D’Ambra, Claudio Ricci, Laura Alberici, Margherita Minghetti, Davide Grego, Virginia Cavallaro, Riccardo Casadei

**Affiliations:** ^1^ Division of Pancreatic Surgery IRCCS Azienda Ospedaliero Universitaria di Bologna Bologna Italy; ^2^ Department of Internal Medicine and Surgery (DIMEC) Alma Mater Studiorum University of Bologna S.Orsola‐Malpighi Hospital Bologna Italy

**Keywords:** long‐term survival, pancreatectomy, pancreatic cancer, pancreatic ductal adenocarcinoma, predictive factors

## Abstract

**Background:**

Long‐term survivors (LTSs) after pancreatic resection of pancreatic ductal adenocarcinoma (PDAC) represent a particular subgroup of patients that remains poorly understood. The primary endpoint was to identify clinicopathological factors associated with LTSs after pancreatic resection for PDAC.

**Methods:**

This was a retrospective study of patients who had undergone pancreatic resection for PDAC. Long survival was defined as a patient who survived at least 60 months. Patients were divided in two groups: LTS and short‐term survivor (STS). The two groups were compared regarding epidemiological, clinical, and pathological data. Propensity score matching (PSM) was used to reduce selection bias with a 1:2 ratio. Multivariable analysis of significative predictive factors before and after PSM was done.

**Results:**

Three hundred and thirty‐three patients were enrolled: 46 (13.8%) in the LTS group and 287 (86.2%) in the STS group. Using PSM, 138 patients were analyzed: 46 in the LTS group and 92 in the STS group. At the multivariate analysis of significative predictive factor after PSM, adjuvant chemotherapy, well‐differentiated tumors (G1), and R0 status were related to long‐term survival (*p* = 0.052, 0.010 and *p* = 0.019, respectively). Kaplan–Meier survival curves confirmed these data. Additionally, Kaplan–Meier survival curves showed that pathological stage I was a favorable factor with respect to stage II, III, and IV.

**Conclusions:**

Long‐term survival is possible after pancreatic cancer resection, even if in a small percentage. Significant predictors of long‐term survival are administration of adjuvant chemotherapy, American Join Committee on Cancer stage I, well‐differentiated tumor (G1), and R0 resection.

## 1 INTRODUCTION

Pancreatic ductal adenocarcinoma (PDAC) is a rare but highly lethal malignancy. In Italy, according to the latest Associazione Italiana Registri Tumori data, around 14.500 new diagnoses are estimated in 2022.^1^ PDAC is the fourth cause of death in women and the sixth in men, representing one of the most ominous prognosis neoplasms, with a 5‐year survival of 11% in males and 12% in females.^1^ Late diagnosis and the lack of effective nonsurgical treatments contribute to the poor prognosis of this disease. In the early stage, surgical resection is currently the only effective treatment, but only 5%–25% of patients are eligible for surgical resection at the time of diagnosis.^2^ Therefore, early diagnosis and timely treatment are currently the only strategies we have to improve the prognosis. Unfortunately, despite the improvements in surgery‐related mortality and morbidity, the post‐resection prognosis remains poor. The majority of the patients affected by PDAC die within the first 2/3 years after surgical resection due to disease recurrence and metastatic spread, with a 5‐year actuarial survival of 20%–25% and effective survival of 10%–20%.^3^ Thus, long‐term survivors (LTSs) after pancreatic resection of PDAC are rare, representing a particular subset of patients that remains poorly understood, mainly in relation to the difficulties to analyze the prognostic factors related to the long survival. The aim of this study is to identify the LTSs and the favorable prognostic factors associated with long‐term survival after pancreatic resection in PDAC in a single center experience.

## 2 MATERIALS AND METHODS

### 2.1 Study design

This was a retrospective study, based on a prospectively maintained database of patients who underwent pancreatic resection for PDAC at Sant’Orsola Hospital, University of Bologna from January 2002 to December 2018. Invasive intraductal papillary mucinous neoplasms (IPMN) or PDAC associated to IPMN were excluded from the study.

After obtaining Ethical Committee of Sant'Orsola Hospital approval (PANBO trial, code: 064/2017/U/OSS) and patient informed consent, the following data were collected for each patient: 1‐relevant variables (sex, age, Eastern Cooperative Oncology Group [ECOG] performance status scale,^4^ American Society of Anesthesiologists [ASA] score, body mass index [BMI], co‐morbidities, and Charlson Comorbidity Index [CCI]^5^); 2‐preoperative data (symptoms, diabetes, jaundice, weight loss, Ca 19‐9, carcinoembryonic antigen, Hb, albumin, creatinine, and bilirubin); 3‐treatment data (neoadjuvant therapy, adjuvant chemotherapy, resectability of the primary tumor according to National Comprehensive Cancer Network,^6^ type of resection, and portal vein resection); 4‐postoperative outcomes (Clavien–Dindo classification,^7^ Clavien–Dindo ≥3, reoperation rate, clinically relevant postoperative pancreatic fistula,^8^ and red blood cell transfusion); 5‐pathological data (tumor size, pathological tumor, node, metastasis [pTNM], grading, perineural invasion, lymph‐vascular invasion, lymph nodes ratio, and R status). Regarding R status, pathology analysis included the evaluation of seven margins: anterior, posterior, superior mesenteric/portal vein groove, superior mesenteric artery, bile duct, pancreatic neck, and stomach margins.

Relevant variables were considered for propensity score matching (PSM) analysis taking into account only those that are related to the death of the patients who underwent pancreatic resection: sex (male and female have a different length of life); age (elderly people more frequently can die for other reasons with respect to young people; thus, usually, elderly lives less time than young people); ECOG Performance Status (PS), ASA score, CCI, presence of comorbidity, and BMI are related to the risk of death of the patient.

The long survivors (LTSs) were defined considering the actual survival, which is the effective survival of the patients. Thus, long survivor was defined as surviving at least 5 years (60 months) from the initial diagnosis.

Patients included in the study were divided into two groups: Long‐Survival Group (LTS) and Short‐Survival Group (STS). Inclusion criteria for LTS group were age >18 years, pancreatic resection, histological diagnosis of PDAC, follow‐up period ≥5 years. The clinic‐pathological characteristics of these two groups were compared and analyzed. Primary endpoints of the study were to identify the percentage of LTS and to evaluate predictive factors related to long‐time survival in patients with PDAC that underwent pancreatic resection. The secondary endpoint of the study was a survival analysis of predictive factors identified.

### 2.2 Statistical analysis

All parameters were reported as frequencies and percentage, mean, median, and standard deviation.

A PSM analysis^9^ was conducted to balance the relevant variables (sex, age, ECOG PS, BMI, CCI, ASA Score and presence of comorbidities). The PSM was performed using logistic regression. The PSM method is closest to the neighborhood method having a caliper width of 0.20 pooled standard, and the matching was created with a 1:2 ratio. Standardized mean difference ,SMD (*d*‐value) was used to assess the balance between the two groups. A *d*‐value <0.2 indicates a small difference between the two groups, identifying an excellent balance. A *d*‐value between 0.2 and 0.5 shows a medium difference, implying a good balance. A *d*‐value between 0.5 and 0.8 shows a high difference, implying a sub‐optimal balance. A *d*‐value >0.8 resulted in a remarkable difference, suggesting a poor balance between the two groups. A multivariate analysis was applied before and after PSM analysis.

Statistical analysis was carried out using Fisher's exact test, Student's *T* test, and Pearson Chi‐square test. SMD (*d*‐value) was used to assess the two group's differences.

Finally, a survival analysis was conducted before and after PSM analysis. Overall survival (OS) was described using the Kaplan–Meyer curves and using Cox proportional hazard models. Survival data were expressed using hazard ratio (HR) and its confidence interval.

The measure of uncertainty was calculated as 95% confidence intervals (95% CI). A two‐tailed *p*‐value inferior to 0.05 was considered statistically significant. Data analysis was performed using Stata/SE, version 18.

## 3 RESULTS

**FIGURE 1:**
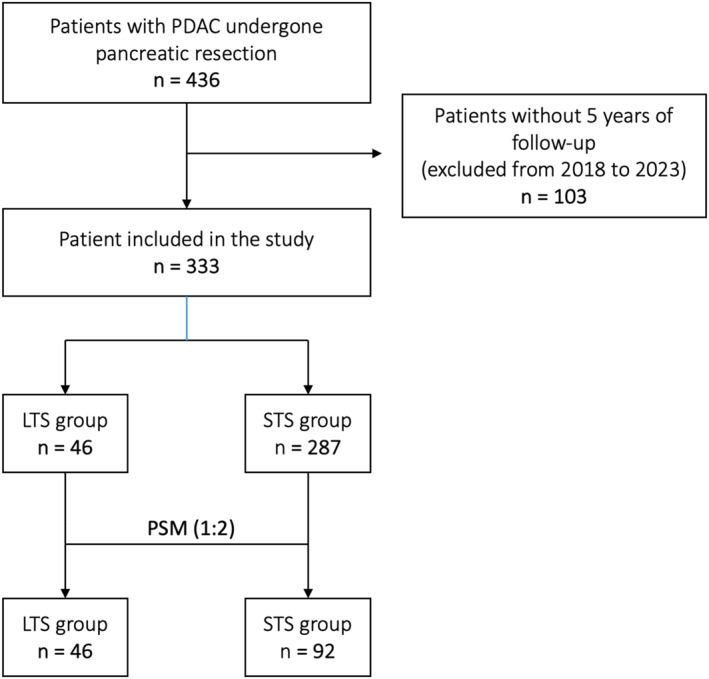
Flowchart of the selection process. LTS, long‐term survivor; PDAC, pancreatic ductal adenocarcinoma; PSM, propensity score matching; STS, short‐term survivor.

The flowchart of the selection process of the patients is summarized in Figure 1. The mean and median follow‐up of the entire population (*n* = 333) were 16.5 months (1.2–161.6) and 28.2 months (±34.6), respectively. The mean and median follow‐up of long‐survivor patient (*n* = 46) were 94.2 months (60.8–190.8) and 100.5 months (±33.8), respectively.

Table 1 summarizes the relevant variables of all the patients included in the study, and the comparison between STS and LTS groups before and after PSM. After PSM 1:2, two groups of 46 and 92 patients did not show suboptimal or poor balancing.

**TABLE 1:** Relevant variables before (unmatched patients) and after (matched patients) propensity score matching.

Relevant variables	Total (*n* = 333)	Unmatched patients				Matched patients			
		LTS (*N* = 46)	STS (*N* = 287)	*p* value	SMD	LTS (*N* = 46)	STS (*N* = 92)	*p* value	SMD
Sex				0.634	*d* = 0.108			0.809	*d* = 0.043
M	166 (49.8)	25 (54.4)	142 (49.5)			25 (54.4)	48 (52.2)		
F	167 (50.1)	21 (45.6)	145 (50.5)			21 (45.6)	44 (47.8)		
Age (yrs ± SD)	68.6 (33.7–88.7)	66.6 (41.4–86.6)	69.5 (33.8–88.7)	**0.051**	*d* = 0.311	66.6 (41.4–86.6)	65.5 (35.4–84.0)	0.781	*d* = 0.051
ECOG PS				**0.075**	*d* = 0.018			0.062	*d* = 0.015
0	146 (43.8)	23 (50)	123 (42.9)			23 (50.0)	37 (40.2)		
1	151 (45.3)	15 (32.6)	136 (47.4)			15 (32.6)	48 (52.2)		
2	31 (9.3)	8 (17.4)	23 (8)			8 (17.4)	6 (6.5)		
3	5 (1.5)	0 (0)	5 (1.7)			0 (0.0)	1 (1.1)		
ASA score				0.597	*d* = 0.168			0.801	*d* = 0.115
1	2 (0.6)	0 (0)	2 (0.7)			0 (0)	0 (0)		
2	102 (30.6)	17 (37)	85 (29.6)			17 (36.9)	30 (32.6)		
3	203 (61.0)	27 (58.7)	176 (61.3)			27 (58.7)	56 (60.9)		
4	26 (7.8)	2 (4.3)	24 (8.4)			2 (4.4)	6 (6.5)		
BMI (kg/m^2^)	24.1 (16.2–43.2)	24.6 (17.2–37.3)	23.9 (16.2–43.2)	0.261	*d* = 0.179	24.6 (17.2–37.3)	24.6 (16.4–43.2)	0.835	*d* = 0.037
Comorbidity				0.626	*d* = 0.226			0.465	*d* = 0.126
No	40 (12.0)	4 (8.7)	36 (12.5)			4 (8.7)	5 (5.4)		
Yes	293 (88.0)	42 (91.3)	251 (87.5)			42 (91.3)	87 (94.6)		
CCI	6 (2–14)	6 (2–12)	6 (3–14)	**0.081**	*d* = 0.276	6 (2–12)	6 (3–14)	0.557	*d* = 0.107

*Note:* Data are expressed as *n* (%), medians value. Bold values provided in this table wants to put in evidence the significant results and the results that show a trend.

Abbreviations: ASA score, American Society of Anesthesiologists score; BMI, body mass index; CCI, Charlson Comorbidity Index; ECOG PS, Eastern Cooperative Oncology Group performance status; LTS, long term survivors; PSM, propensity score matching; STS, short term survivors; SMD, standardized mean difference.

Table 2 summarizes the comparison between the two groups regarding preoperative data, type of treatment, postoperative, and pathological data before and after PSM.

**TABLE 2:** Univariate analysis of potential predictive factors before (unmatched patients) and after (matched patients) propensity score matching analysis.

Predictive factors	Total (*n* = 333)	Unmatched patients				Matched patients			SMD
		LTS (*n* = 46)	STS (*n* = 287)	*p* value	SMD	LTS (*n* = 46)	STS (*n* = 92)	*p* value	
Preoperative Ca 19.9	33 (0.6–2885.2)	13 (1.2–2000)	181 (0.6–2885.2)	0.206	*d* = 0.201	13 (1.2–2000)	34.2 (0.6–2885.2)	**0.088**	*d* = 0.127
Preoperative CEA	2.7 (0–35.1)	2.3 (0–35.1)	2.95 (0.5–34.2)	0.823	***d* = 0.036**	2.3 (0–35.1)	4 (0.7–34.2)	0.390	*d* = 0.221
Preoperative Hb	12.5 (8.5–16.8)	12.7 (8.7–16.8)	12.4 (8.2–16.6)	0.183	*d* = 0.212	12.7 (8.7–16.8)	12.6 (9.1–16.6)	0.545	*d* = 0.117
Preoperative albumin	39 (16–50)	40 (22–50)	38 (16–49)	0.363	*d* = 0.145	40 (22–50)	39 (22–49)	0.817	*d* = 0.046
Preoperative creatinine	0.8 (0.3–8.4)	0.8 (0.5–1.6)	0.8 (0.3–8.4)	0.264	*d* = 0.178	0.8 (0.5–1.6)	0.8 (0.3–8.4)	0.385	*d* = 0.139
Preoperative bilirubin	1.4 (0.2–35)	0.6 (0.2–29)	1.8 (0.2–35)	**0.060**	*d* = 0.300	0.6 (0.2–29)	1.3 (0.3–35)	**0.079**	*d* = 0.327
Symptoms				**0.017**	*d* = 0.438			**0.039**	*d* = 0.383
No	106 (31.8)	22 (47.8)	84 (29.3)			22 (47.8)	27 (29.4)		
Yes	227 (68.2)	24 (52.2)	203 (70.7)			24 (52.2)	65 (70.6)		
Diabetes				0.597	*d* = 0.142			0.550	*d* = 0.122
No	239 (71.8)	35 (76.1)	204 (71.1)			35 (76.1)	65 (70.7)		
Yes	94 (28.2)	11 (23.9)	83 (28.9)			11 (23.9)	27 (29.3)		
Jaundice				**0.006**	*d* = 0.512			**0.032**	*d* = 0.401
No	160 (48.0)	31 (67.4)	129 (44.9)			31 (67.4)	44 (47.8)		
Yes	173 (52.0)	15 (32.6)	158 (55.1)			15 (32.6)	48 (52.2)		
Weight loss				**0.064**	*d* = 0.473			0.255	*d* = 0.229
No	252 (75.7)	40 (87.0)	212 (73.9)			40 (87.0)	72 (78.3)		
Yes	81 (24.3)	6 (13.0)	75 (26.1)			6 (13.0)	20 (21.7)		
Neoadjuvant CT				0.397	*d* = 0.212			0.301	*d* = 0.198
No	306 (91.9)	41 (89.1)	265 (92.3)			41 (89.1)	87 (94.6)		
Yes	27 (8.1)	5 (10.9)	22 (7.7)			5 (10.9)	5 (5.4)		
Adjuvant CT				0.188	*d* = 0.312			**0.050**	*d* = 0.261
No	92 (27.6)	9 (18.6)	83 (28.9)			9 (18.6)	33 (35.9)		
Yes	241 (72.4)	37 (81.4)	204 (71.1)			37 (81.4)	59 (64.1)		
Resectability of primary				0.932	*d* = 0.016			1.000	*d* = 0.024
Resectable	249 (74.8)	34 (73.9)	215 (74.9)			34 (73.9)	67 (72.8)		
Borderline resectable	79 (23.7)	12 (26.1)	67 (23.3)			12 (26.1)	25 (27.2)		
Locally advanced	5 (1.5)	0 (0)	5 (1.8)			0 (0)	0 (0)		
Type of resection				**0.050**	*d* = 0.232			**0.077**	*d* = 0.174
PD	162 (48.7)	24 (52.2)	138 (48.1)			24 (52.2)	48 (52.2)		
DP	75 (22.5)	15 (32.6)	60 (20.9)			15 (32.6)	17 (18.5)		
PT	96 (28.8)	7 (15.2)	89 (31.0)			7 (15.2)	27 (29.3)		
SMV/PV resection				0.318	*d* = 0.312			0.255	*d* = 0.229
No	267 (80.2)	40 (87.0)	227 (79.1)			40 (87.0)	72 (78.3)		
Yes	66 (19.8)	6 (13.0)	60 (20,9)			6 (13.0)	20 (21.7)		
C‐D classification ≥3				1.000	*d* = 0.004			1.000	*d* < 0.001
No	253 (75.9)	35 (76.1)	218 (76.0)			35 (76.1)	70 (76.1)		
Yes	80 (24.1)	11 (23.9)	69 (24.0)			11 (23.9)	22 (23.9)		
CR‐POPF				0.696	*d* = 0.125			0.820	*d* = 0.082
No	265 (79.6)	38 (82,6)	227 (79.1)			38 (82.6)	73 (79.4)		
Yes	68 (20.4)	8 (17.4)	60 (20.9)			8 (17.4)	19 (20.6)		
RBD transfusion				0.744	*d* = 0.102			1.000	*d* = 0.023
No	206 (61.9)	30 (65.2)	176 (61.3)			30 (65.2)	59 (64.1)		
Yes	127 (38.1)	16 (34.8)	111 (38.7)			16 (34.8)	33 (35.9)		
Reoperation				0.649	*d* = 0.089			1.000	*d* < 0.001
No	288 (86.5)	39 (84.8)	249 (86.8)			39 (84.8)	78 (84.8)		
Yes	45 (13.5)	7 (15.2)	38 (13.2)			7 (15.2)	14 (15.2)		
Tumor size (mm)	30 (7–100)	25 (8–70)	30 (7–100)	0.118	*d* = 0.249	25 (8–70)	30 (8–70)	0.554	*d* = 0.112
Pathological TNM stage				**0.003**	*d* = 0.602			**0.010**	*d* = 0.641
I	85 (25.5)	21 (45.7)	64 (22.3)			21 (45.7)	20 (21.7)		
II	136 (40.8)	18 (39.0)	118 (41.1)			18 (39.0)	37 (40.2)		
III	101 (30.3)	7 (15.2)	94 (32.7)			7 (15.2)	33 (35.9)		
IV	11 (3.3)	0 (0)	11 (3.8)			0 (0)	2 (2.2)		
Grading				**<0.001**	*d* = 0.670			**0.003**	*d* = 0.646
G1	48 (14.4)	14 (30.4)	34 (11.9)			14 (30.4)	12 (13.0)		
G2	179 (53.8)	27 (58.7)	152 (52.9)			27 (58.7)	48 (52.2)		
G3	106 (31.8)	5 (10.9)	101 (35.2)			5 (10.9)	32 (34.8)		
Perineural invasion				**0.023**	*d* = 0.415			0.148	*d* = 0.283
No	135 (40.5)	26 (56.5)	109 (38.0)			26 (56.5)	39 (42.4)		
Yes	198 (59.5)	20 (43.5)	178 (62.0)			20 (43.5)	53 (57.6)		
Lymph‐vascular invasion				0.270	*d* = 0.286			0.291	*d* = 0.214
No	250 (75.1)	38 (82.6)	212 (73.9)			38 (82.6)	68 (73.9)		
Yes	83 (28.9)	8 (17.4)	75 (26.1)			8 (17.4)	24 (26.1)		
LN ratio	0.14 (0.0–1.0)	0.0 (0.0–0.75)	0.14 (0.0–1.0)	**0.001**	*d* = 0.511	0.0 (0–0.75)	0.13 (0–0.72)	**<0.001**	*d* = 0.699
R status				**0.004**	*d* = 0.563			**0.010**	*d* = 0.536
R0	179 (53.8)	34 (73.9)	145 (50.5)			34 (73.9)	46 (50.0)		
R1	154 (46.2)	12 (26.1)	142 (49.5)			12 (26.1)	46 (50.0)		
Year of diagnosis				0.746	*d* = 0.059			1.000	*d* < 0.001
2002–2009	209 (62.8)	30 (65.2)	179 (62.4)			30 (65.2)	60 (65.2)		
2010–2018	124 (37.2)	16 (34.8)	108 (37.6)			16 (34.8)	32 (34.8)		

*Note*: Data are expressed as *n* (%) and medians value. Bold values provided in this table wants to put in evidence the significant results and the results that show a trend.

Abbreviations: C‐D, Clavien–Dindo; CEA, carcinoembryonic antigen; CR‐POPF, clinically‐relevant post‐operative pancreatic fistula; CT, chemotherapy; DP, distal pancreatectomy; Hb, hemoglobin; LN ratio, lymph‐node ratio, is the number of lymph‐nodes involved/the number of lymph‐nodes removed; LTS, long term survivors; PD, pancreaticoduodenectomy; PSM, propensity score matching; PT, portal trunk; SMD, standardized mean difference; SMV/PV, superior mesenteric vein/portal vein; STS, short term survivors; TNM, tumor, node, metastasis; TP, total pancreatectomy.

In the unmatched population, preoperative bilirubin levels were higher in STS than in the LTS group but not statistically significant (1.8 vs. 0.6 mg/dL; *p* = 0.060). Symptoms were more frequently present in STS than LTS (70.7% vs. 52.2%; *p* = 0.017) as well as jaundice (55.1% vs. 32.6%; *p* = 0.006) and weight loss (26.1% vs. 13.0%; *p* = 0.064). Types of pancreatic resections were significantly different: pancreaticoduodenectomy (PD) and distal pancreatectomy (DP) were more frequently performed in the LTS group than in STS (52.2% and 32.6% vs. 48.1% and 20.9%, respectively), whereas total pancreatectomy was performed more frequently in the STS group than in LTS (31.0% vs. 15.2%; *p* = 0.050). Pathological TNM stage, grading, perineural invasion, lymph nodes ratio, and R status were significantly different between the two groups compared.

In the matched population, low preoperative Ca 19‐9 and bilirubin serum value showed a trend toward LTS (*p* = 0.088 and 0.079, respectively). The presence of symptoms and jaundice was significantly more frequent in STS than LTS (*p* = 0.039 and 0.032, respectively). Adjuvant chemotherapy was more frequently performed and completed in LTS than in STS (*p* = 0.050). The pathological TNM stage was significantly different between two groups; in particular, stage I was more frequent in the LTS group (45.7% vs. 21.7%) and stage III was more frequent in the STS group (39.1% vs. 15.2%) (*p* = 0.010). The lymph‐node ratio was confirmed to be higher in the STS group (*p* < 0.001). Well‐differentiated (G1) tumors rate was significantly higher in the LTS group (30.4% vs. 13.0%) and high grade (G3) tumors rate was significantly higher in the STS group (34.8% vs. 10.9%) (*p* = 0.003). R0 resection, compared with R1 resection was more frequently found in the LTS group (73.9% vs. 50.0%, *p* = 0.010) than in STS. Interestingly, neoadjuvant chemotherapy was not related to long‐term survival.

Table 3 showed the multivariate analysis of significative predictive factors before and after PSM. The independent factors related with LTS were adjuvant chemotherapy (*p* = 0.052), grading (*p* = 0.010), and R status (*p* = 0.019).

**TABLE 3:** Multivariable analysis of significative predictive factors before and after PSM.

Significative predictive factors	Before PSM			After PSM		
	OR	*p*‐value	95% IC	OR	*p*‐value	95% IC
Age	1.03	0.742	0.88; 1.20	1.00	0.977	0.92; 1.09
ECOG PS	3.07	0.146	0.68; 13.91	2.35	0.107	0.83; 6.65
CCI	0.37	0.193	0.08; 1.66	0.97	0.906	0.61; 1.54
Preoperative Ca 19.9	0.99	0.104	0.99; 1.00	1.00	0.627	1.00; 1.00
Preoperative bilirubin	1.09	0.275	0.93; 1.27	1.03	0.596	0.93; 1.13
Symptoms	0.81	0.846	0.10; 6.69	1.05	0.950	0.20; 5.54
Jaundice	0.21	0.248	0.01; 3.00	0.39	0.265	0.08; 2.03
Weight loss	1.00	–	–	1.35	0.696	0.30; 6.01
Adjuvant chemotherapy	0.47	0.383	0.09; 2.54	4.48	**0.052**	0.98; 20.34
Type of resection	0.91	0.877	0.29; 2.92	0.96	0.902	0.48; 1.90
Pathological TNM stage	0.85	0.798	0.25; 2.94	0.52	0.203	0.19; 1.43
Grading	0.11	**0.008**	0.02; 0.55	0.24	**0.010**	0.08; 0.72
Perineural invasion	0.28	0.109	0.06; 1.32	0.53	0.288	0.17; 1.70
LN ratio	0.05	0.348	0.00; 23.96	0.19	0.503	0.01; 22.86
R status	0.10	**0.041**	0.01; 0.91	0.20	**0.019**	0.05; 0.77

Abbreviations: CCI, Charlson Comorbidity Index; ECOG PS, Eastern cooperative oncology group performance status; LN ratio, lymph‐node ratio, is the number of lymph‐nodes involved/the number of lymph‐nodes removed; PSM, propensity core matching; TNM, tumor, node, metastasis.

**FIGURE 2:**
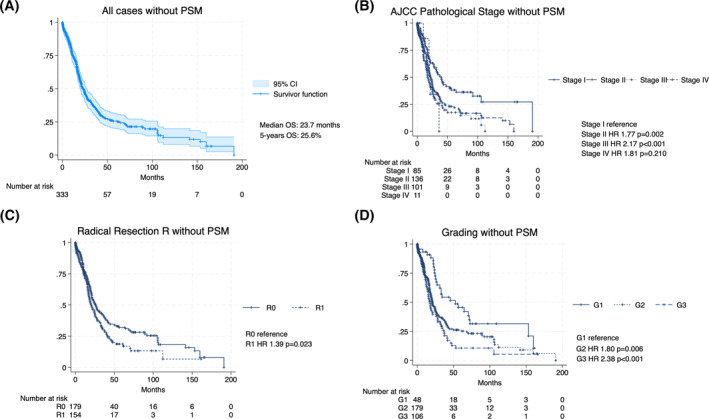
Survival analysis of all patients (*n* = 333) included in the study before PSM analysis: (A) survival analysis of all patients and regarding, (B) AJCC pathological stage, (C) R status, and (D) grading. AJCC, American Join Committee on Cancer.

**FIGURE 3:**
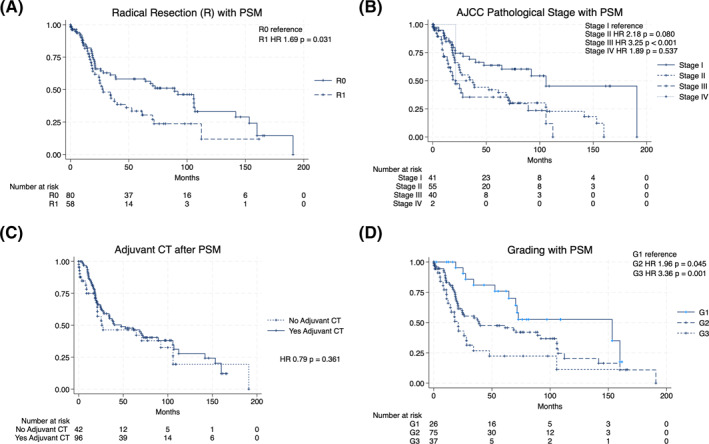
Survival analysis of patients included in the study after PSM analysis: (A) survival analysis regarding R status, (B) AJCC pathological stage, (C) adjuvant chemotherapy, and (D) grading. AJCC, American Join Committee on Cancer.

Survival analysis showed that the overall 5‐years survival was 25.6%, with a median OS of 23.7 months (Figure 2A). The survival analyses showed, before PSM analysis, a significant improvement of the OS of patients with well‐differentiated (G1), compared to moderately (G2) and poorly differentiated (G3) PDAC (HR 1.80, *p* = 0.006 and HR 2.38, *p* < 0.001) (Figure 2D). Similarly, patients with a lower pTNM stage had significantly better long‐term survival rates than those with higher pTNM stage, in particular, stage I versus stage II (HR = 1.77, *p* = 0.002) and stage II versus stage III (HR 2.17, *p* < 0.001) (Figure 2B). Finally, patients with negative resection margins had higher survival rates than patients with R1 status (HR 1.39, *p* = 0.023) (Figure 2C). After PSM analysis, grading, R status, and pTNM were confirmed as factors related with LTS (Figure 3). In particular, G1 was significantly related to LTS if compared with G2 (HR = 1.96; *p* = 0.045) and G2 with G3 (HR = 3.36; *p* = 0.001) (Figure 3D). R0 was related to LTS if compared with R1 (HR = 1.69, *p* = 0.031) (Figure 3A). Pathological stage I was related to LTS if compared with stage II (HR = 2.18; *p* = 0.080) and stage II with stage III (HR = 3.25; *p* < 0.001) (Figure 3B). On the contrary, adjuvant chemotherapy was not related with LTS (HR = 0.79; *p* = 0.361) (Figure 3C).

## 4 DISCUSSION

Several studies have analyzed the prognostic factors of long‐term survival and evaluated the 5‐year survival in patients affected by PDAC who underwent pancreatic resection,^10^, ^11^, ^12^, ^13^, ^14^, ^15^, ^16^, ^17^, ^18^ but only few studies compared LTS and STS.^19^, ^20^

The present study has some peculiar characteristics: 1‐it compared patients affected by PDAC who underwent pancreatic resection with a follow‐up of at least 5 years; 2‐survival was calculated as actual survival, which is the effective survival of the patients; 3‐the LTS and STS groups were compared and PSM analysis was done to equate the two groups using relevant variables related to the probability of death.

Our study showed that, among 333 patients resected for PDAC with a follow‐up ≥5 years, a small percentage (*n* = 46, 13.8%) of patients affected by PDAC who underwent pancreatic resection were alive after a follow‐up of at least 5 years (LTS). This datum shows how, in this subset of patients affected by PDAC, long‐term survival is possible even if in a small percentage of cases.

Additionally, the present study identified, at multivariate analysis after PSM, three favorable factors related to LTS after the surgical resection of PDAC: well‐differentiated tumors (G1), R0 resection, and adjuvant chemotherapy. Survival analysis showed the best OS in patients with well‐differentiated tumors (G1) in pathological stage I who performed a R0 resection.

By analyzing these data, it is evident that the most important prognostic factors related to long‐term survival seem to be those related to the pathological characteristics of the tumor and those in which pancreatic resection is performed carefully with an adequate lymphadenectomy and obtaining an R0 resection. Due to these data, it is clear how an early diagnosis and a properly performed pancreatic resection can be considered the most important factors to obtain a long‐term survival.

Regarding pTNM, stage I was more frequent in the LTS group and stage III in STS the group (*p* = 0.010). Stage I includes all the tumors <2 cm (T1) and between 2 and 4 cm (T2), without lymph nodes (N0) and distant metastasis (M0). For the *T* parameter, different size thresholds have been proposed, the most consensual being 20 mm. Garcea et al.^21^ reported that this cutoff value had the best impact on OS. In addition, Takahashi et al.^22^ suggested that the tumor size may not be directly responsible for a poor survival outcome but may rather reflect more aggressive biological behavior being associated with other adverse pathological characteristics (perineural, vascular invasion, poor differentiation, and positive resection margins). Considering these data, it could be reasonable to suggest that the tumor size might be used as a selecting factor to detect the most appropriate candidates for surgery and to suggest a neoadjuvant treatment in potentially resectable tumors.

Regarding tumor grade, the association between well‐differentiated tumor and longer survival is well‐known.^18^, ^23^, ^24^, ^25^, ^26^, ^27^ In this study, well‐differentiated tumors (G1) were significantly more frequent in the LTS group with respect to STS (30.4% vs. 13.0%; *p* = 0.003), whereas poorly differentiated tumors in STS with respect to LTS (34.8% vs. 10.9%). These data means that 1‐ surgery could be proposed when resectable PDAC is well‐differentiated; it could be avoided, and neoadjuvant treatment is preferred, when PDAC is poorly differentiated and 2‐ preoperative biopsy has to be performed to allow a proper treatment.

Finally, a highly significant prognostic factor is the effectiveness of surgery and in particular the state of the surgical margins after resection.^18^, ^28^ Richter et al.^29^ identified the R0 resection margin as the only post‐surgical prognostic factor. In our previous study^28^ we suggested that, among all the histologically evaluated resection margins, the superior mesenteric artery margin seemed to be the most important one to define both R status and disease‐free survival. In this study, the percentage of R0 resection was significantly higher in the LTS group than in STS (73.9% vs. 50.0%; *p* = 0.010) and it represents an independent factor related to LTS. These data mean that the primary goal of surgery with a curative intent is to achieve an R0 resection. Thus, pancreatic resection has to be performed carefully and preferably in a tertiary referral center by an experienced surgeon.

Other reported prognostic factors were not considered independent, but they were significant or represent a trend in univariate analysis after PSM analysis: preoperative levels of cancer (CA) 19.9, presence of symptoms, jaundice, type of resection, and lymph‐node ratio. In particular, the elevation of CA19‐9 seems to predict tumor recurrence.^30^ In addition, in Ca 19‐9 nonsecretors, the prognostic value of Ca125 should be routinely assayed in surgical candidates.^31^ These data could be considered in a large cohort of patients and they can suggest to perform surgery up‐front in resectable, asymptomatic patients with low Ca 19‐9.

Interestingly, in this study, neoadjuvant chemotherapy was not associated with improved outcomes (at 5 years). In consideration of the great relevance of this information, we want to underline two aspects of this results: (1) in the present study, it is probably related to the small sample of patients who underwent neoadjuvant chemotherapy and to the fact that the new protocol of chemotherapy or chemoradiotherapy was rarely adopted; (2) in general, we have to consider the evolution of surgical and oncologic care in the last 2 decades.^32^, ^33^, ^34^, ^35^, ^36^, ^37^ Actually, in fact, neoadjuvant chemotherapy has an important role in the treatment of patients with borderline resectable and locally advanced cancer (it can allow surgical resection in a relevant proportion of patients), but in resectable pancreatic cancers, its role is controversial. The role of mini‐invasive pancreatic surgery was not evaluated because we do not perform mini‐invasive PD and the data could be not reliable. However, mini‐invasive DP can be safely implemented because it is considered feasible, safe, and oncologically equivalent technique for pancreatic cancer as well as open approach.^38^, ^39^, ^40^ This study has some limitations. The retrospective design in a single center, the long duration of the study (2002–2018), and the lack of molecular and genetics data. In particular, the long period of the study is related with the development of the treatment strategies, surgical techniques, and perioperative mortalities. In addition, the new protocol of chemotherapy or chemoradiotherapy was rarely adopted as well as the neoadjuvant approach.^41^, ^42^, ^43^, ^44^

In conclusion, by analyzing the subset of patients affected by PDAC who underwent pancreatic resection, we found some favorable factors for long‐term survival, referred to pathological characteristics of the tumor (pTNM stage I, well‐differentiated tumors), in patients in which pancreatic resection is performed carefully obtaining an R0 resection and it is followed by the administration of adjuvant therapy.

Other prognostic factors can be considered, even if they are not independent factors (symptoms, jaundice, and serum Ca 19‐9) to identify those patients with an early metastatic potential prior to intervention. Indeed, these patients do not benefit from surgery, and other strategies (i.e., neoadjuvant chemotherapy) are likely to be more appropriate. In summary, these factors seems to be reliable factors to predict tumor evolution and to indicate the best treatment for each patients affected by resectable PDAC. However, the results of the present study need to be confirmed in further and well‐structured studies including genetic and molecular factors with an adequate study of tumor immune microenvironment that could be very important to understand long‐term survival better.

## AUTHOR CONTRIBUTIONS

**Carlo Ingaldi**: Writing—original draft. **Vincenzo D'Ambra**: Data curation. **Claudio Ricci**: Conceptualization. **Laura Alberici**: Validation. **Margherita Minghetti**: Data curation. **Davide Grego**: Data curation. **Virginia Cavallaro**: Methodology. **Riccardo Casadei**: Writing—original draft.

## CONFLICT OF INTEREST STATEMENT

The authors declare no conflicts of interest.

## ETHICS STATEMENT

All procedures performed in studies involving human participants were in accordance with the ethical standards of the institutional and/or national research committee and with the 1964 Helsinki declaration and its later amendments or comparable ethical standards.
